# 

**Published:** 1877-05

**Authors:** 


### Table of Contents.

### ORIGINAL COMMUNICATIONS.

Sulphate of Cinchonidia and Sulphate of Quinine. *By Wm. A. Love, M.D., Atlanta, Ga.* .......... 65

Adulterated Medicine. *By Dr. J. S. Pemberton, Atlanta, Ga.* .......... 71

### REPORTS OF SOCIETIES.

Atlanta Academy of Medicine .......... 74

### SELECTIONS.

Phosphorus—Its use in Medicine .......... 88
Septicæmia after Amputations .......... 95
Subacute Pneumatic Arthritis .......... 103
On the Best Means of Promoting Union by First Intention .......... 105

### EDITORIAL.

Medical Association of Georgia .......... 108
Tax on Professions .......... 111
Editorial Correspondence .......... 112, 115
The Discovery and Discoverers of Anæsthesia .......... 116
Death from Chloroform .......... 119
Antihydropin .......... 120
Notices .......... 120

### BIBLIOGRAPHICAL.

Atlas of Skin Diseases .......... 121
Elementary Treatise on Diseases of the Skin .......... 122
What is the Comparative Physiological and Therapeutical Action of Free Phosphorus and the Phosphites? .......... 122
Contributions to Reparative Surgery .......... 123
Transactions of the Pathological Society of Philadelphia .......... 123
Transactions of American Gynæcological Society .......... 123
Solution and Absorption of Medicines .......... 123

### EXCERPTA.

Relief of Pain in Uterine Cancer .......... 124
The Lateral Movements in the Knee-Joint in Cases of White Swelling 125
Insufflation as an Auxiliary Method in Surgical Operations .......... 126
Symptoms Resulting from Anæsthesia by Ether in Young Subjects .. 126
Kentucky State Medical Society .......... 127
Hypodermic Injection in Hernia .......... 127
Ergot in Chronic Uterine Catarrh .......... 128
Esmarch's Operation for Closed Jaw .......... 128

